# A Hybrid convolution neural network for the classification of tree species using hyperspectral imagery

**DOI:** 10.1371/journal.pone.0304469

**Published:** 2024-05-31

**Authors:** Jian Wang, Yongchang Jiang

**Affiliations:** School of Management, Harbin University of Commerce, Harbin, 150028, China; University of Electronic Science and Technology of China, CHINA

## Abstract

In recent years, the advancement of hyperspectral remote sensing technology has greatly enhanced the detailed mapping of tree species. Nevertheless, delving deep into the significance of hyperspectral remote sensing data features for tree species recognition remains a challenging endeavor. The method of Hybrid-CS was proposed to addresses this challenge by synergizing the strengths of both deep learning and traditional learning techniques. Initially, we extract comprehensive correlation structures and spectral features. Subsequently, a hybrid approach, combining correlation-based feature selection with an optimized recursive feature elimination algorithm, identifies the most valuable feature set. We leverage the Support Vector Machine algorithm to evaluate feature importance and perform classification. Through rigorous experimentation, we evaluate the robustness of hyperspectral image-derived features and compare our method with other state-of-the-art classification methods. The results demonstrate: (1) Superior classification accuracy compared to traditional machine learning methods (e.g., SVM, RF) and advanced deep learning approaches on the tree species dataset. (2) Enhanced classification accuracy achieved by incorporating SVM and CNN information, particularly with the integration of attention mechanisms into the network architecture. Additionally, the classification performance of a two-branch network surpasses that of a single-branch network. (3) Consistent high accuracy across different proportions of training samples, indicating the stability and robustness of the method. This study underscores the potential of hyperspectral images and our proposed methodology for achieving precise tree species classification, thus holding significant promise for applications in forest resource management and monitoring.

## Introduction

Environmental protection is a core focus of current societies across the globe. The classification of forest tree species is important for the protection of trees, as well as for the supervision of large forests. Tree species recognition can be broadly classified into traditional artificial and remote sensing approaches [1]. The former requires more manual input and material resources compared to the latter, as well as a great deal of investigation time. Furthermore, traditional methods can also only be performed on a single object, resulting in extensive monitoring periods [2]. Manual measurements may not be possible in harsh environments. Remote sensing technology can overcome these limitations, allowing for the regional monitoring of large areas, rapid observations and short repetition periods at a low cost. Remote sensing approaches are commonly applied in current forest classification research [3]. As an important source of remote sensing data, hyperspectral imaging has become increasingly popular. However, the multiple bands of the hyperspectral imagery can result in computationally expensive analysis procedures due to the huge amount of data.

In recent years, with the rapid development of computer hardware and algorithms, deep learning methods based on neural networks have been widely applied in various fields [4]. As an emerging research direction in the field of machine learning, deep learning utilizes deep neural network structures to automatically learn high-level abstract features and then combines these features layer by layer to achieve efficient and accurate data classification and prediction [5–9]. Compared to traditional machine learning methods, deep learning possesses stronger adaptability and generalization capabilities, enabling better handling of large-scale complex data, and has achieved tremendous success in fields such as computer vision, natural language processing, and speech recognition. In the field of remote sensing, deep learning technology has attracted extensive attention from scholars, and many experts have utilized deep learning methods for tree species classification, achieving good classification results [10–12]. Particularly, convolutional neural networks (CNNs) have achieved significant success in computer vision tasks such as image classification, object detection, and semantic segmentation [13–15]. Due to their powerful feature extraction capabilities, CNNs have become the most commonly used neural networks in hyperspectral tree species classification [16–18].

Classification methods based on spectral features have been employed in tree species classification. Xi et al. applied 1D-CNN to classify tree species using OHS-1 hyperspectral images, revealing that the accuracy achieved with 1D-CNN (85.04%) exceeded that of the random forest classification model (80.61%). Nevertheless, 1D-CNN solely accounts for the spectral information of samples, disregarding their spatial information, leading to moderate classification performance [19,20].

The spatial information, which does not change following the dimension reduction, determines the spatial characteristics of adjacent pixels. Spatial information features can compensate for the lack of spectral features and the application of spatial characteristics can improve the classification of hyperspectral data. PCA dimensionality reduction was conducted on hyperspectral data and employed 2D-CNN to extract spatial features from the reduced dataset [21]. They successfully classified seven dominant species and dead trees in mixed coniferous forests, achieving an accuracy rate of 87%. However, despite utilizing pixel spatial information, the 2D-CNN lost the original spectral information during the dimensionality reduction process.

Classification methods that leverage the relationship between spatial and spectral features are pivotal for tree species classification. One approach entails employing 3D-CNN to concurrently extract spectral and spatial features of pixels [22]. An enhanced 3D convolutional neural network was proposed for tree species classification, utilizing raw data from aerial hyperspectral images as input, without necessitating dimensionality reduction or feature selection [23]. This model can extract spectral and spatial features simultaneously, achieving a commendable classification accuracy of 93.14%. Nonetheless, this method solely relies on a 3D convolutional structure, which may result in overfitting when the number of network parameters is substantial. Alternatively, distinct networks can be employed to extract spectral and spatial features independently, subsequently amalgamating them for classification [24]. For example, a Spectral-Spatial Parallel Convolutional Neural Network (SSPCNN) was introduced for classifying forest tree species in unmanned aerial vehicle hyperspectral imagery [25]. Experimental researches demonstrate that SSPCNN surpasses other methodologies. However, this network structure, although effective, may not be optimal for intricate forest environments.

Previous studies have demonstrated the feasibility of CNN for the identification and classification of tree species with high-dimensional data. However, 2D-CNN is not able to effectively extract the identification features from the spectral dimensions. Complex shortcomings, such as a lack of features or model information, can reduce the classification accuracy for hyperspectral data. 3D-CNN is able to extract cube features within hyperspectral data to improve recognition accuracy by including spectral dimensions into the convolution calculations. However, the increase in dimensions of HIS inevitably intensifies the time complexity and degradation of the algorithm, particularly for spectral bands of similar textures. Thus, in the paper, we combine a hybrid CNN model with a support vector machine (SVM) classifier in order to overcome the shortcomings of the previous models. Hybrid-CS was trained for tree species classification and included sample sets generated from HJ-1A data. The model makes full use of spectral and spatial feature maps to achieve the maximum accuracy.

## 2. Materials and methods

### 2.1 Study area

**Fig 1:**
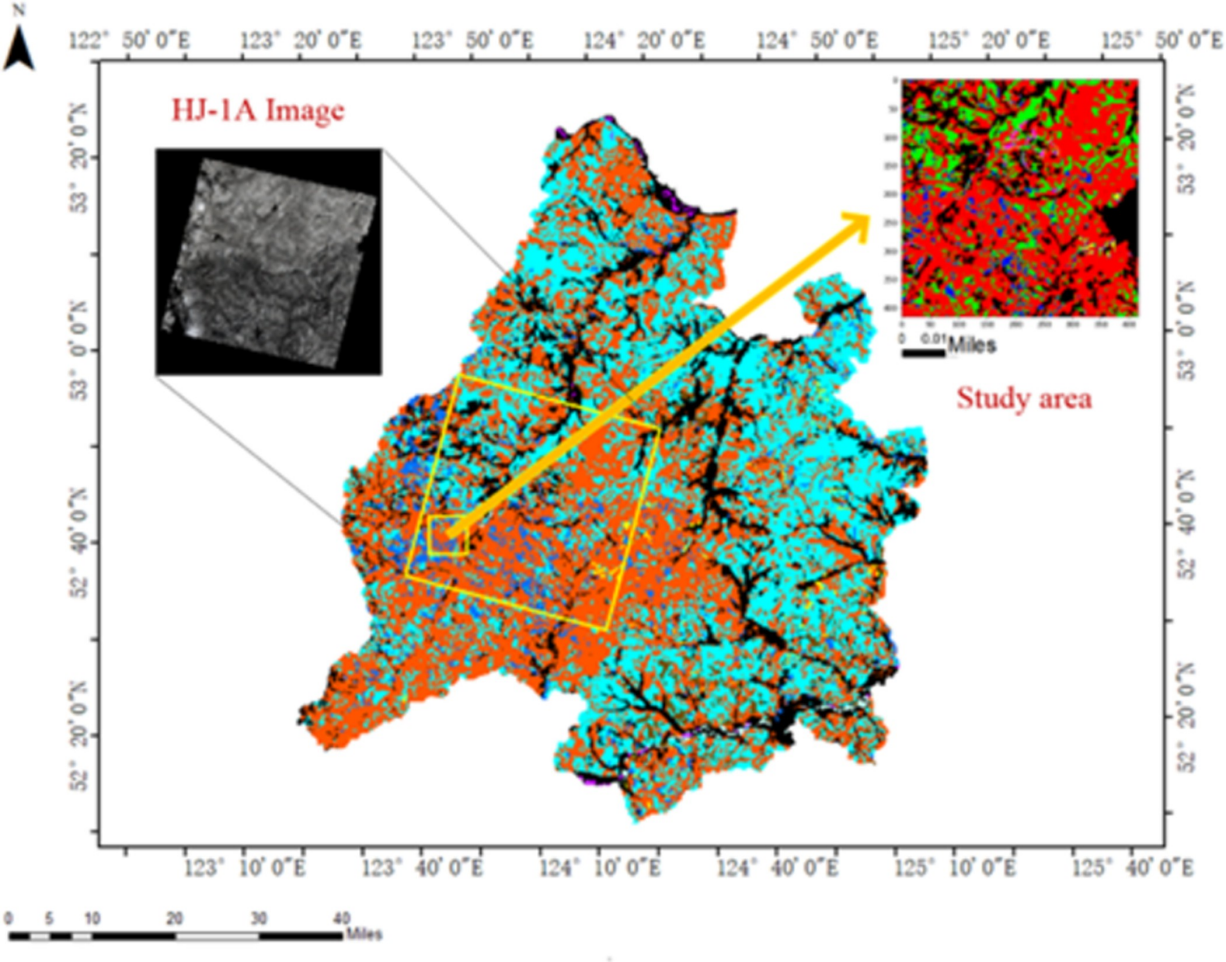
Map of study area.

The study area is situated within the jurisdiction of the Tahe Forestry Bureau (Fig 1), nestled amidst the Daxing’an Mountains in the northwestern region of Heilongjiang Province, China (between 123° to 125° E and 52° to 53° N). Encompassing a border length of 173 km and spanning a total area of 14,420 km^2^, these areas experience a cold-temperate continental climate marked by notable climatic variations. Summers are characterized by brief periods of hot and humid weather, while winters are long, dry, and bitterly cold. The average annual temperature in the area stands at -2.4°C, with an average yearly precipitation of 463.2 mm, primarily concentrated in July and August. Forested areas, which occupy 81% of the total land area and boast a timber stockpile of 53.4 million m^3^, dominate the landscape. The prevailing tree species include birch, larch, spruce, mongolica, willow, and poplar [4].

### 2.2 Dataset

To classify tree species, we utilized data sourced from the HJ-1A and Sentinel-2 satellites. Fig 1 illustrates the Hyperspectral Imaging (HSI) data from HJ-1A obtained from the China Center for Resources Satellite Data and Application. The HJ-1A satellite boasts a high-speed imaging system featuring 115 bands and a spatial resolution of 100 m, providing comprehensive data for coastal and land remote sensing [17]. To address the relatively low resolution of the HSI data, we utilized ENVI 5.1 software to enhance the resolution of the HJ-1A/HSI images collected on 20 August 2016 to match the MSI spatial resolution. The interpolation method was employed to resample the experimental HSI data. In 2018, the Tahe Forestry Bureau conducted a survey and utilized the results to classify major forest species in the research region. The study areas were delineated into 500 × 500 × 115 pixels for HSI data. We selected the area with the highest species diversity as the research focus, which encompassed Birch, Larch, Spruce, Mongolia, Willow, and Poplar. Table 1 outlines the three study areas utilized in this study, with the training samples comprising approximately one-third of the total samples.

**Table 1:** List of 6 tree species samples of the study area.

Tree Species	Birch	Larch	Mongolia	Poplar	Spruce	Willow
Study Area	130,124	39,216	57,620	3019	15,330	3492

### 2.3 Hybrid-CS model

Meaningful spectral features and spatial features are extracted from the data source by the Hybrid C-S model. The desired spectral features and spatial features are extracted to enable all HSI bands to jointly reconstruct the feature mapping, thereby minimizing spectral distortion. Compared to other networks, Hybrid C-S model has lower complexity because it requires less computational resources.

**Fig 2:**
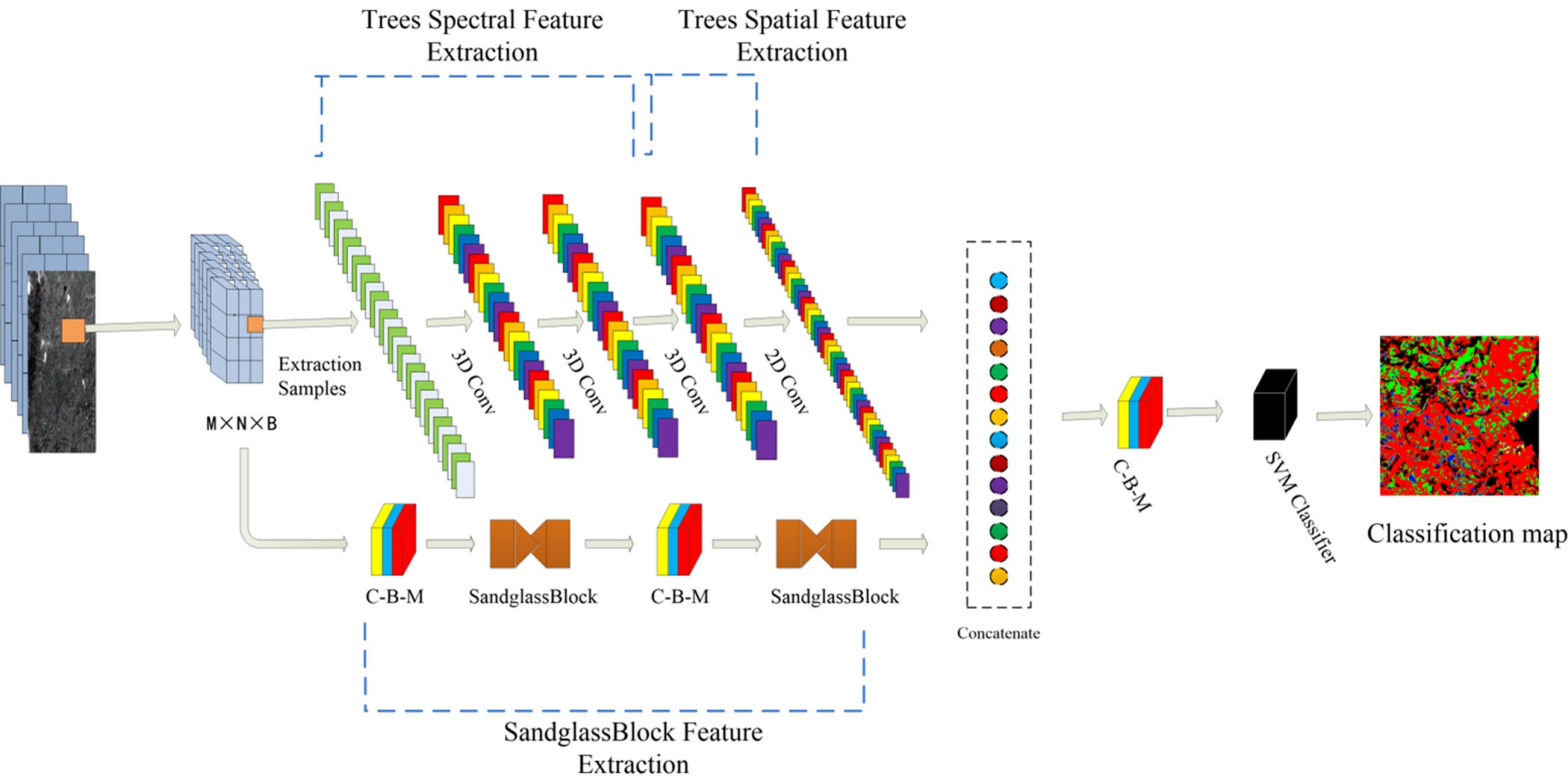
Hybrid-CS HSI tree classification model.

The proposed Hybrid C-S model is illustrated in Fig 2. The spectral information of pixels is determined by their average reflectance spectra, while spatial features are associated with neighboring pixels. In this framework, the spectral branch extracts spectral and spatial features, while the sandglass module branch extracts advantageous features; they are then fused for subsequent classification. To minimize the complexity of tree classification and maintain sample imbalance, we adopt a framework that integrates multiple features from the spectral branch and the sandglass branch to complete the classification process.

#### 2.3.1 SS feature extraction

The dimension of hyperspectral image data is presented as Eq (1), where M×N and Q represent the spatial size and spectral bands of HSI, respectively. This is a three-dimensional structure that contains rich spatial and spectral information.


$$I\epsilon R^{M\times N\times Q},$$ (1)

Where, I is the original input, M is the width, N is the height, and Q is the number of spectral bands (depth). Each pixel in hyperspectral image (HSI) denoted as I, contains Q spectra and constitutes the vector as described in Equation:

$$Y=\left(y_{1},y_{2},\ldots ,y_{c}\right)\in R^{1\times 1\times c}$$ (2)

Where, c represents the tree species (there are 7 species of tree species in this paper, so c = 7). However, hyperspectral pixels can contain a mixture of tree species, making it difficult for any model to introduce the intra-class variability and inter-class similarity of hyperspectral pixels into I. In order to minimize spectral redundancy, principal component analysis (PCA) was employed to reduce the dimensions of the original HSI data. PCA was applied consistently across the same space dimensions (width and height of M and N, respectively) and time domain, resulting in a reduction of spectral bands from Q to P, thereby significantly decreasing computational complexity. The PCA reduced data cube is denoted as *X∈R*^*M*×*N*×*P*^, where X represents the input data post PCA dimension reduction.

Image recognition technology segments the hyperspectral data into small three-dimension cubes, where the category of each cube is determined by the label of the central pixel. Eqs (3) and (4) describe the 2D- and 3D-CNN, respectively. We established spatial points p∈R^(C×C×D) centred at (α,β), with a width and height of C, covering D bands. The number of cubes is calculated as (M-C+1)×(N-C+1). Consequently, the cubes cover a width from α-((C-1))⁄2 to α+((C-1))⁄2 and a height from β-((C-1))⁄2 to β+((C-1))⁄2, with the bands reduced from 95 to 30 by PCA.

$$v_{i,j}^{x,y}=g\left(\sum \sum_{p=0}^{P_{i-1}}\sum_{q=0}^{Q_{j-1}}w_{i,j}^{p,q}v_{i}^{\left(x+p)(y+q\right)}+b_{i,j}\right)$$ (3)

$$v_{i,j}^{x,y,z}=g\left(\sum \sum_{p=0}^{P_{i-1}}\sum_{q=0}^{Q_{j-1}}\sum_{r=0}^{R_{m-1}}w_{i,j,m}^{p,q,r}v_{(i-1)m}^{\left(x+p)(y+q)(z+r\right)}+b_{i,j}\right)$$ (4)

where $v_{i,j}^{x,y}$ is the activation value at the spatial position (x,y) from the i-th layer, which corresponds to the j-h feature of the map, i is the ordinal number of the neural network, j is the ordinal number of the characteristic samples. *P*_*i*_ and *Q*_*i*_ are the length and width of the convolution kernel in two-dimensional space, *R*_*m*_ is the height of the convolution kernel in the third dimension; m represents the input data from the upper layer, $v_{i,j}^{x,y,z}$ is the value of the point at position (x,y,z), $w_{i,j,m}^{p,q,r}v_{(i-1)m}^{\left(x+p)(y+q)(z+r\right)}$ represents the weight of (p,q,r) from the m-th feature, *b*_*i*,*j*_ represents the offset of the i-th layer from the j-th feature maps, and g(x) signifies the activation function. The 2D-CNN input data are convoluted via a 2D convolution kernel by computingthe sum of the products between the input data and convolution kernel. The convolution kernel is applied across a fixed window over the input data to cover the entire three-dimensional cube (HSI) [26]. Convolution features are then extracted from the nonlinear activation function.

The 2D-CNN acts as a two-dimension filter to extract spatial features, where P and Q can be applied as convolution windows to extract features from a single band. The 3D-CNN is able to extract the features from the R bands via the convolution windows of sizes P and Q by increasing the convolution kernel from 2D to 3D. In Fig 2, R equals 3 and the convolution kernel dimensions are P×Q×3. The 3D-CNN can thus combine both spatial and spectral information.While the SVM classifier is typically employed to optimize parameters by minimizing cross-entropy in the convolutional neural network, experiments have shown the SVM classifier to be more effective in parameter optimization by maximizing the maximum interval classification hyperplane when replacing the Softmax output layer of the original CNN. In the process of hyperspectral tree species recognition, capturing both spectral and spatial information of the tree species is essential, in addition to extracting two-dimensional classification features from the spatial dimensions. While the 2D-CNN can process spatial information only, the 3D-CNN kernel can extract both spectral and spatial features maps of the tree species from HSI data, albeit with increased computational complexity.

#### 2.3.2 Sandglass block extraction

**Fig 3:**
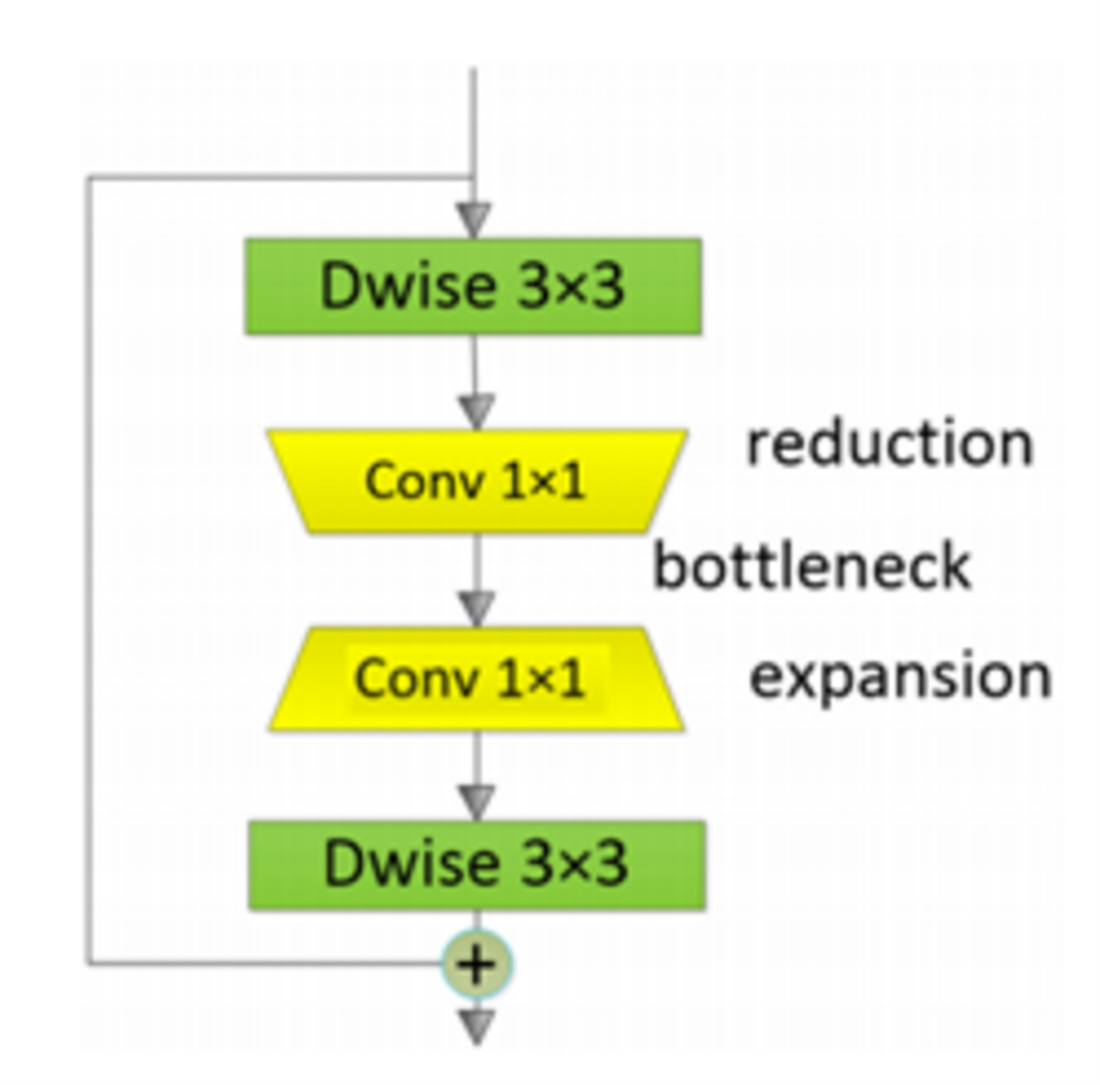
Sandglass block with bottleneck structure.

The purpose of the Sandglass block is to minimize parameters and computational costs by flipping residual down sampling. The Sandglass block represents the corresponding relative channel numbers with the thickness of each block, as shown in Fig 3. Residual blocks are constructed inversely between bottlenecks, with depth separable convolutions (separation blocks) included at both ends of the residual path. To preserve information from the lower layers and facilitate gradient propagation across layers when transitioning to the top layers, we locate shortcuts to connect high-dimensional representations. As depth convolutions are relatively lightweight, high-dimensional features can be used to encode richer spatial information and generate more expressive representations.

Sandglass feature extraction module includes two layers of C-B-M and two layers of Sandglass block. Then the SS features and Sandglass features are concatenated into the classification module. Sandglass feature extraction module was constructed by two C-B-M layers and two Sandglass layers. C-B-M consists of a Convolutional layer, followed by a Batch Normalization layer, and finally a Smish activation function layer [27].

#### 2.3.3 Feature fusion

After the original data is processed through the spectral-spatial extraction branch feature F_SS_ and the Sandglass branch feature F_sand_ are obtained separately. We combine these two types of features to further utilize the multi-feature information of the data. Since the F_SS_ and F_sand_ are in different domains, a concatenation operation is chosen instead of an addition operation to maintain the independence of the two features. These features are merged to form an F_SS_ feature.. The two mechanism methods are fused with the traditional SVM algorithm for the tree species classification using hyperspectral image. Multiple weights are assigned to the original features of HS data so that each feature is recognized to the greatest extent possible. Redundant features are removed before the subsequent classification experiment. The formula is calculated as follows:

$$F_{\left(ss,sand\right)}=concatencate\;\left(F_{ss}⊕F_{sand}\right)$$ (5)

The mechanism formula provides the new weight, which is assigned to the corresponding features, where, ss denotes the spectral-spatial value, sand denotes the value of sandglasss block, and *F*_(*ss*,*sand*)_ denotes the concatenated features of the three equations. Finally, ‘⊕’ denotes the operation of the feature fusion algorithm. The fusion information concatenated with the output of the provides rich, detailed features for subsequent classification work.

#### 2.3.4 Evaluation indicators

Generally in the classification of hyperspectral remote sensing images, the classification result babel is compared with the real feature information one by one. The overall accuracy (OA), average accuracy (AA) and Kappa coefficient (kappa) were determined using Eqs (6)–(8), respectively in order to evaluate the classification of the approach proposed in this paper.


$$OA=\frac{\sum_{i=1}^{K}C(i,i)}{M}$$ (6)

$$AA=\frac{\sum_{i=1}^{K}OA}{K}$$ (7)

$$Kappa=\frac{M\sum_{i=1}^{K}C(i,i)-\sum_{i=1}^{K}(C(i,+)C(+,i))}{M^{2}-\sum_{i=1}^{K}(C(i,+)C(+,i))}$$ (8)

Where, C represents a confusion matrix, Assuming that the sample has k types, C∈R^k×k^, and M represents the number of training samples, C_(i,i)_ represents the number of samples that are correctly classified into the i-th category.

#### 2.3.5 Comparison methods

To demonstrate the superiority and effectiveness of the proposed method in this study, we compared it with traditional machine learning methods such as 3D-CNN, as well as other state-of-the-art deep learning methods such as 3D-CNN, DBMA, DBDA, DBSSN. Next, we will briefly introduce the methods compared.

1. 3D-CNN: Three-Dimensional Convolutional Neural Network. The specific network architecture is based on the 3D-CNN [28], and the input data size is 1 × 7 × 7 bands, where “band” represents the number of spectral bands, and 7 denotes patch size.
2. DBMA: Dual-branch multi-attention network. The specific network architecture is based on a dual-branch structure, dense blocks, CBAM attention mechanism [29], and input data size consistent with 3D-CNN.
3. DBDA: Double-Branch Dual-Attention Network. The network architecture, outlined in Li et al., is based on a double-branch structure, dense blocks, and the DANet attention mechanism, with input data size aligned with a 3D-CNN [30].
4. DBSSN: A double-branch spatial–spectral joint model. The network architecture, utilizes two branches to fully leverage the spatial and spectral information of pixels [31]. In the part of feature fusion, the SimAM attention mechanism is incorporated to refine features, extracting crucial characteristics, with input data size set as band × 7 × 7.

## 3. Experiment and discussion

### 3.1 Parameter adjustment

Parameter settings and adjustments are crucial parts of Hybrid-CS testing as they reflect the performance of network. To ensure fairness in experimental results, despite the two types of data having different resolutions, the ground truth maps for multiple data with fixed resolution are given the same treatment. Table 2 describes each layer of the DBMF method. In the experiments, the proposed method and other methods compared are all based on the same ground truth maps.

**Table 2:** The layer types, output map dimensions, and number of parameters of the Hybrid-CS model.

SS Tunnel			Sandglass Tunnel		
Layer	Kernel Size	Output Shape	Layer	Kernel Size	Output Shape
Input		(7 × 7 × 100)	Input		(7 × 7 × 100)
3d-Con	(1 × 1 × 7)	(7 × 7 × 47, 24)	C-B-M	(3 × 3 × 1)	(7 × 7 × 1, 24)
3d-Con		(7 × 7 × 47, 24)	Sandglass Block		(7 × 7 × 1, 24)
3d-Con		(7 × 7 × 47, 24)	C-B-M		(7 × 7 × 1, 24)
2d-Con	(1 × 1 × 47)	(7 × 7, 60)	Sandglass Block	(3 × 3 × 1)	(7 × 7 × 1, 24)
Pooling	(7 × 7 × 1)	(1 × 1, 60)	Pooling	(3 × 3 × 1)	(7 × 7 × 1, 24)
Concatenate		(1 × 60)	Concatenate		(1 × 60)
Layers (Fusion)			Output Shape (Fusion)		
JOINT			(1 × 60)		
C-B-M			(7 × 7 × 1, 60)		
Classifier			(1 × 60)		

The Hybrid-CS model has a complex branch structure, numerous parameters, and requires a long training time, necessitating repeated testing. The input kernel is computed and characterized using OA and AA. When the input kernel is (7 × 7), the network model performs the best, which will be discussed in detail in the next section. According to the desired training performance and network convergence speed, the SGD strategy with a learning rate of 0.001 is also adopted in the network.

### 3.2 Data training

During the training process of the proposed model, the size of the convolutional kernel has a significant impact on classification accuracy. If the convolutional kernel is set to be larger, detailed information may be lost while relevant data is retained. Conversely, if the size is set to be smaller, the opposite may occur. Therefore, the size of the convolutional kernel directly affects the classification results of tree species. In this experiment, the most common convolutional kernel sizes are n, where n = 1, 3, 5, 7, 9, 11, and 13. The model is trained with a batch size of 100 samples over 1000 epochs, and the results are shown in Table 3. From the table, it can be observed that the accuracy is highest when the size is 7, which is suitable for large-scale machine learning operations.

**Table 3:** The AA of kernel size in the experiment.

Kernel size	1	3	5	7	9	11	13
AA	0.64	0.68	0.72	0.91	0.82	0.74	0.63

In order to simultaneously increase the number of spectral and spatial characteristic graphs, the spectral information of the input HSI data is retained in the output data block with the 3D convolution. The 2D convolution is applied prior to the flatten layer because it can distinguish the spatial information of different spectral bands without generating a huge loss of spectral information. In particular, the number of nodes in the last dense layer is equal to the number of tree species (i.e., 7). Therefore, the total number of parameters in the proposed model depends on the number of classes in the dataset. The SVM loss and SGD optimizer are used to randomly initialize and train the tensor values. We use a small batch size of 256 samples to train the model for 50 iterations without batch standardization and data augmentation.

Specifications of the hardware used in the experiments are detailed as follows: Operating system: Ubuntu 18.04.3; GPU:P100-PCIE; CUDA: version 10.1; and memory: 24 G. Experiments were repeatedly performed based on the classification result in order to determine a best learning rate of 0.001.

**Fig 4:**
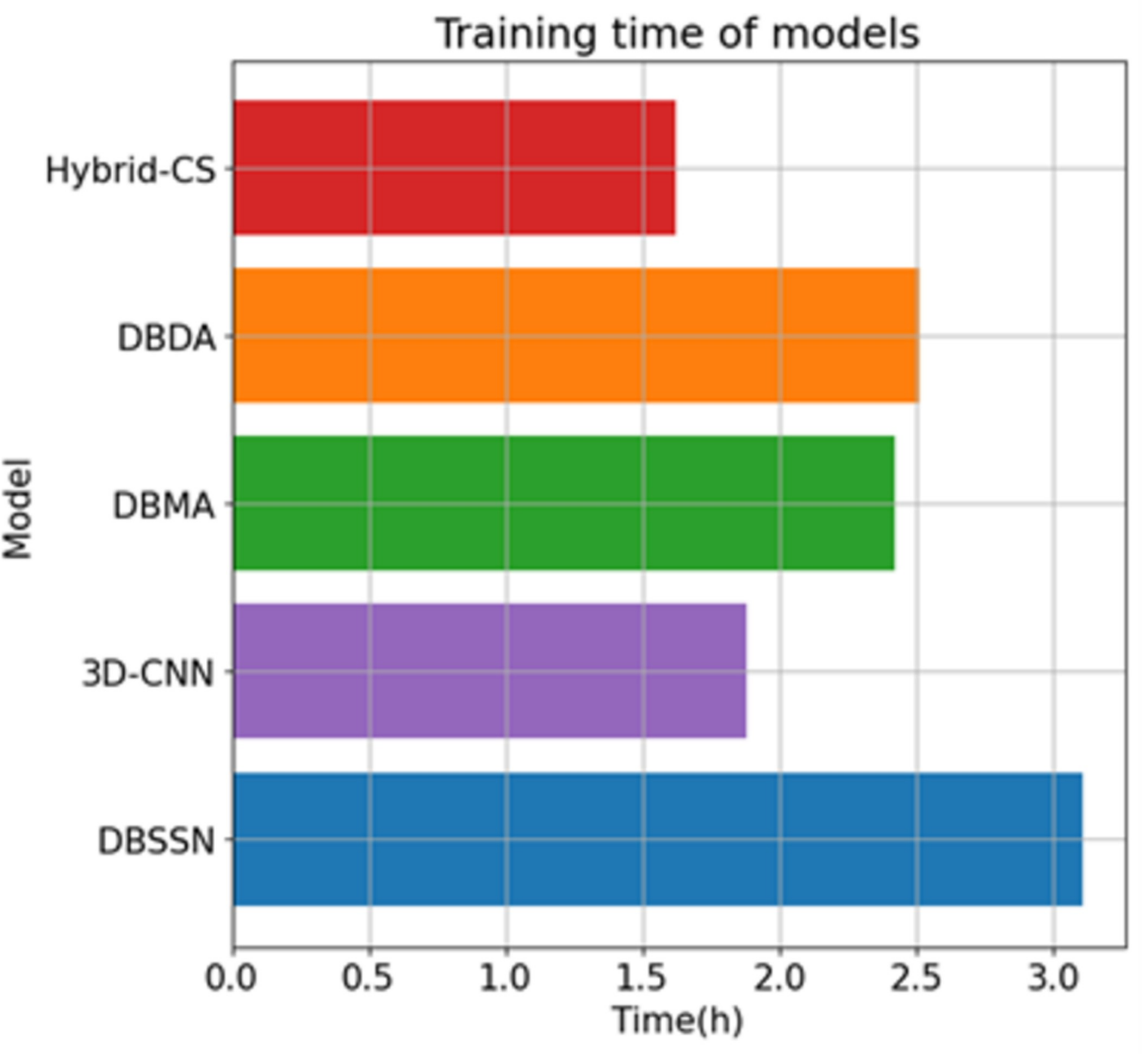
Training time.

The parameter file size for Hybrid-CS, containing all layers, is 310 megabytes. Data training is conducted using stochastic gradient descent. Fig 4 illustrates the training time consumption of the tested models, with Hybrid-CS taking approximately 100 minutes, significantly faster than DBSSN (30 minutes). The 3D-CNN method is characterized by its lightweight design and fast convergence speed, albeit slightly slower compared to Hybrid-CS. Among the compared methods, Hybrid-CS exhibits the fastest learning speed, while DBSSN has the longest learning time. These results indicate that the Hybrid-CS model is the fastest learner and demonstrates superior deep learning capabilities in tree species classification compared to other models.

**Fig 5:**
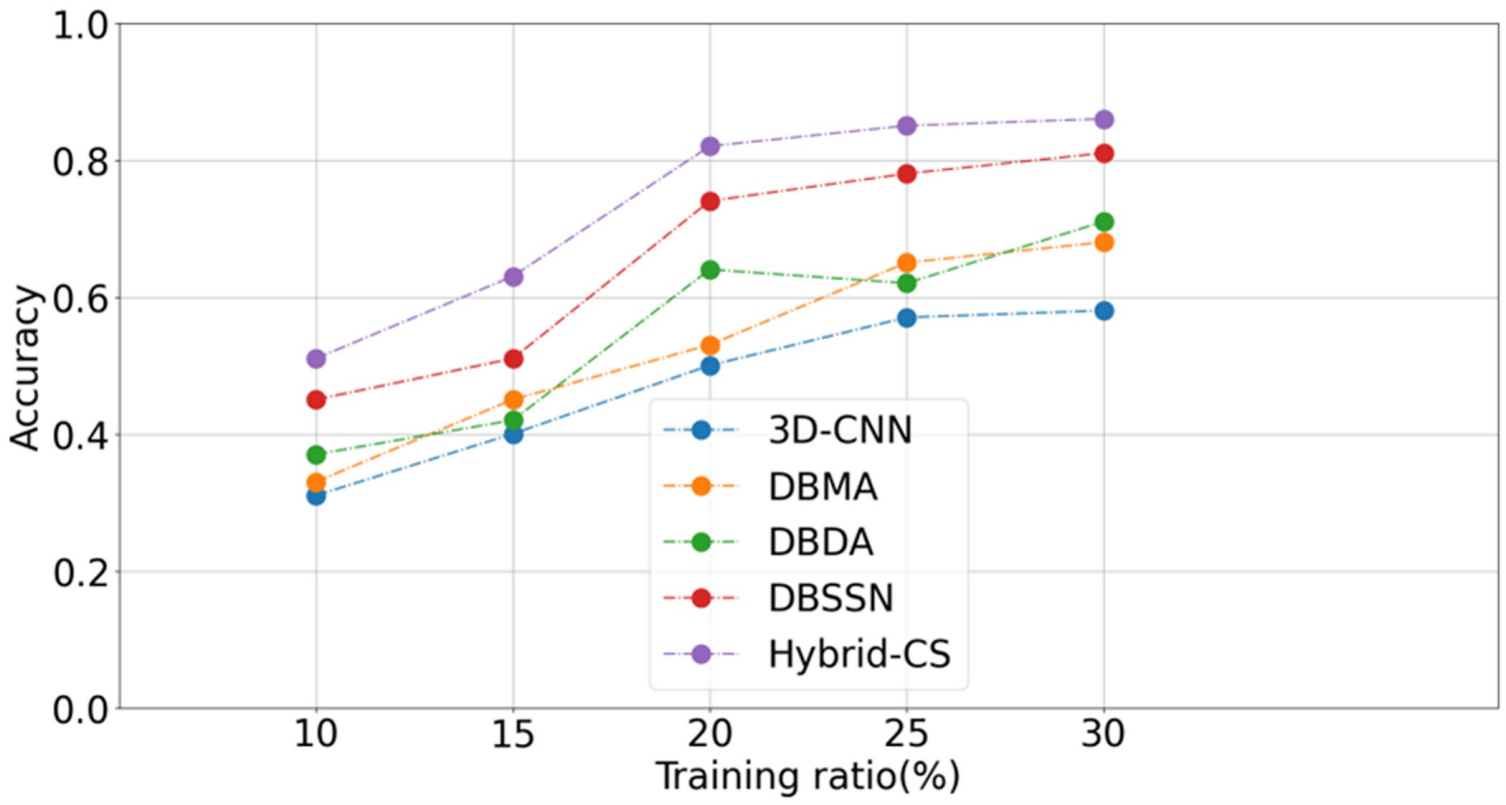
Accuracy with the proportion of training samples.

From Fig 5, it can be observed that the overall accuracy (OA) exhibits a slow and gradual increase with the number of samples. They all share a common trend: the higher the number of training samples, the higher the accuracy. However, at each stage, the proposed method outperforms other methods, indicating its strong capability in learning spatial and spectral features. The OA of the proposed model reaches a peak of 92% when the training samples account for 80%, after which it stabilizes. The accuracies of other algorithms also remain relatively stable but are consistently lower than the Hybrid-CS method proposed in this study.

### 3.3 Classification results

**Fig 6:**
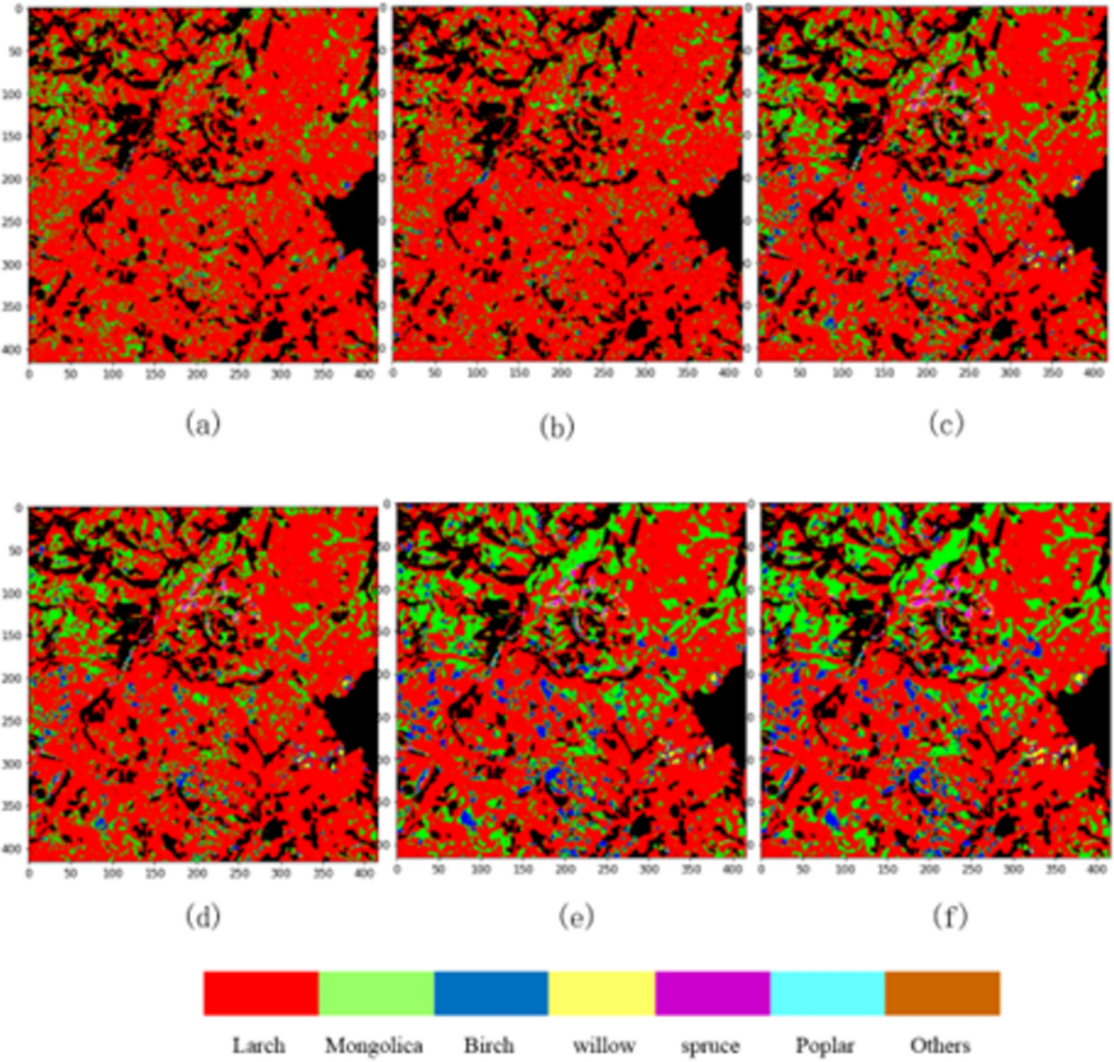
Trees species classification map. (a) 3D-CNN. (b) DBMA. (c) DBDA. (d) DBSSN. (e) Hybrid-CS. (f) Ground truth.

For the comparison between classification approaches, we randomly assigned 30% of the sample to the training group, with the remaining assigned to the test group. Fig 6 presents the results of the classification. Table 4 reports the OA, AA and Kappa values determined by different classification models. The Hybrid-CS outperforms other methods on the same hyperspectral dataset, achieving the accuracy of over 91% while maintaining minimal standard deviation. Initially, Hybrid-CS extracts spectral-spatial feature vectors through a combination of 3D-CNN and 2D-CNN layers. The extracted features are then concatenated with the sandglass block and passed to the SVM algorithm for classification. By integrating these three components, Hybrid-CS successfully classifies the input images. Our research findings indicate the performance trends of each model as follows (from highest to lowest): 3D-CNN, DBMA, DBDA, DBSSN, and Hybrid-CS.

**Table 4:** Classification results of different models (%).

	OA	AA	Kappa
3D-CNN	59.59	60.93	55.89
DBMA	61.69	61.81	59.92
DBDA	70.40	71.72	69.56
DBSSN	87.87	89.11	86.01
Hybrid-CS	91.35	92.67	90.25

Table 5 illustrates the tree species classification maps obtained using different methods on the tree species dataset of the study area. The quality of the classification map produced by Hybrid-CS is notably superior to that of other methods. Additionally, Hybrid-CS demonstrates strong capabilities in tree species identification, achieving an overall accuracy of 91.3%. The accuracy rates for each tree species in Hybrid-CS are as follows: Birch 92.3%, Larch 90.8%, Mongolica 90.2%, Poplar 87.1%, Spruce 85.9%, and Willow 88.2%. While both DBSSN and DBDA methods can extract spectral and spatial information simultaneously, the classification performance of Hybrid-CS surpasses both, yielding results closer to the reference map. Moreover, the spectral and spatial features provided by Hybrid-CS are more robust compared to other methods, resulting in smoother appearances.

**Table 5:** Classification accuracy of tree species with different models (%).

	Larch	Birch	Mongolica	spruce	Willow	Poplar
3D-CNN	68.12	51.89	62.50	58.23	58.99	58.09
DBMA	65.89	55.78	62.39	59.97	64.31	63.35
DBDA	71.55	69.34	68.28	70.11	76.13	72.19
DBSSN	89.11	86.39	88.16	80.32	88.98	85.18
Hybrid-CS	90.82	92.38	90.26	87.97	88.27	87.12

## 4. Discussion

### 4.1 Influence of sandglass block on Hybrid-CS

In Section 2.3.3, the Sandglass block in Hybrid-CS is elucidated. Here, the effectiveness of tree species classification is demonstrated using the tree species dataset. As a crucial parameter, the optimal image patch size for Hybrid-CS is determined to be 7, with other conditions remaining consistent with Section 3.1. Subsequently, the performance of "Complete Hybrid-CS" is compared with that of "Hybrid-CS without the Sandglass block."

**Fig 7:**
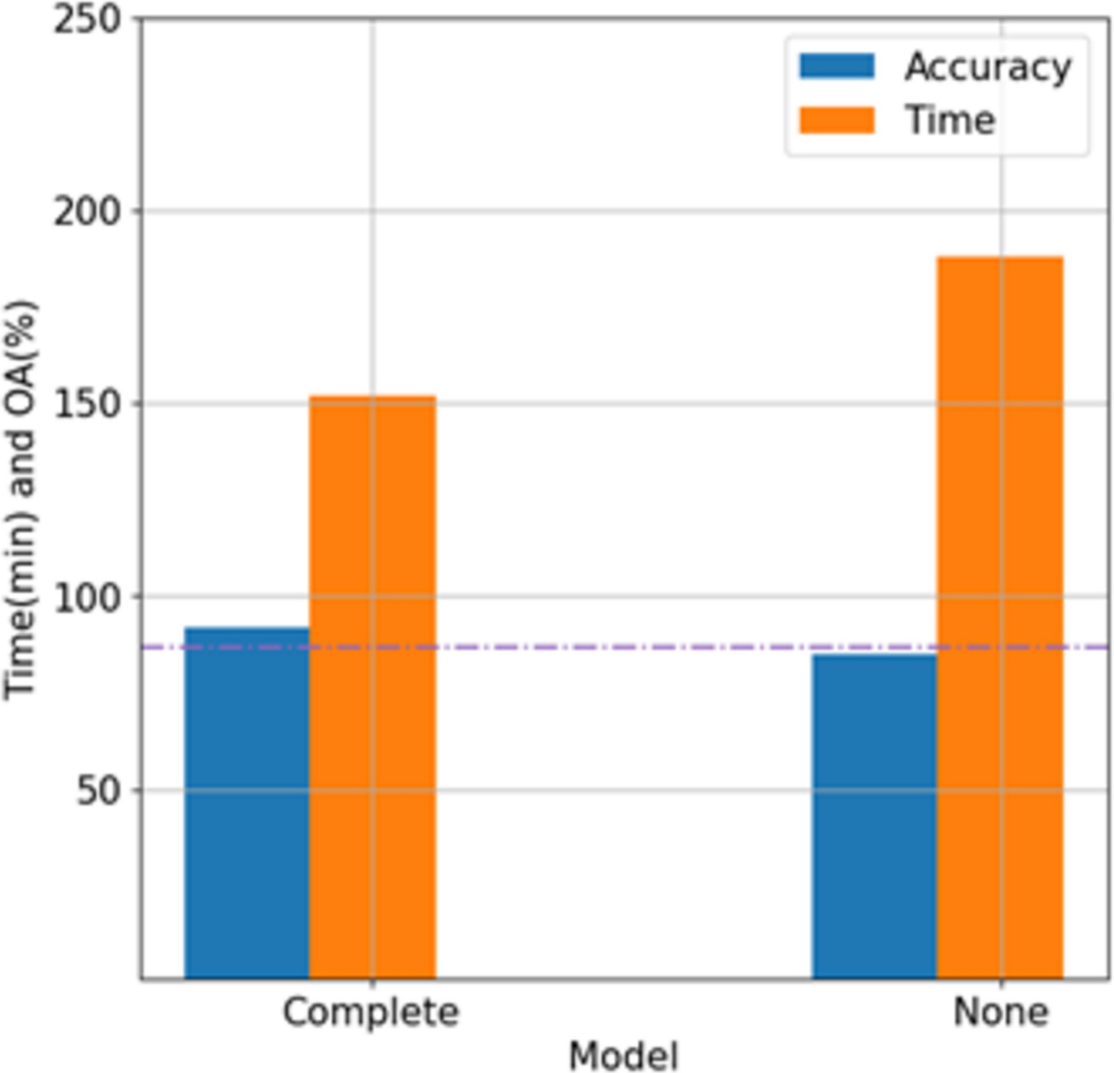
Training time and OA of the Hybrid-CS model with sandglass block and none.

As depicted in Fig 7, Hybrid-CS with the Sandglass block surpasses the one without it. The OA of the dataset improves by nearly 6%, and the training time for the complete dataset is approximately 37 minutes faster than that without the Sandglass block. Since the hourglass block adds shortcut connections between high-dimensional representations and conducts deep convolution in the high-dimensional feature space, it accelerates backpropagation, leading to performance disparities.

### 4.2 Hybrid-CS versus other models for the accuracy of tree species classification

The Hybrid-CS model employs a spectral-spatial fusion mechanism, where the extracted spectral and spatial information not only undergo deep feature selection but also exploit a wealth of deep features. Furthermore, through the joint fusion network, deep features are integrated effectively, reducing redundant features and enhancing feature fusion capability. Similarly, the feature extraction achieved through the Sandglass blocks has been demonstrated in the preceding section. As shown in Section 3, the network’s operational results reveal that the Hybrid-CS based on tree species dataset outperforms other methods in terms of OA, kappa, and AA. Additionally, it excels particularly in identifying spruce and larch, offering commercial value and rarity to the industry. Following Hybrid-CS, DBSSN, DBDA, and DBMA methods rank next, with 3D-CNN exhibiting the poorest performance. Amidst the results characterized by fragmentation, rough edges, low accuracy, and significant mixing of the four smallest tree species, Hybrid-CS demonstrates good recognition of six tree species. Overall, despite deepening the convolutional network layers, Hybrid-CS does not suffer from gradient degradation or overfitting during training, resulting in the best classification outcomes. Consequently, this method can serve as an effective approach for the complex tree species classification in Northeast China.

### 4.3 Dig deep reason for the results

The 3D-CNN method, known for its lightweight design, versatility, and fast convergence, preserves intricate spectral details through its convolution operations, making it suitable for tree classification tasks, especially those with large sample sizes. However, it falls short in meeting the specific requirements of tree species classification. On the other hand, the DBMA method, utilizing 3D-CNN, employs channel attention and spatial perception mechanisms to enhance features. Nevertheless, its training process is hindered by parameter influence, making it more time-consuming than CD-CNN. Additionally, DBMF overlooks the sequence and relationship of HS data, reducing its effectiveness. Consequently, these limitations make DBMA less efficient and effective in tree classification compared to the Hybrid-CS method. Unlike DBMA, DBDA integrates a Mish activation function to extract information from negative parameters, albeit at the cost of increased algorithmic complexity and reduced training efficiency. While this improves accuracy compared to DBMA, it compromises overall performance. DBSSN, grounded in residual learning principles, independently extracts spectral and spatial features before fusion classification. However, it tends to neglect fine crown details, resulting in average classification performance. In contrast, the proposed Hybrid-CS method accelerates training speed through sandglass block without compromising its ability to extract crucial information and maintain connectivity with spatial-spectral features. By forgoing preprocessing or post-processing of HSI data and spatial-spectral information, Hybrid-CS maximizes utility and achieves optimal classification accuracy. Addressing issues of information loss inherent in feature downsizing and selection in original spectral-spatial data, Hybrid-CS offers a promising solution for forestry science management applications.

## 5. Conclusion

This paper introduces a hybrid HSI classification model based on CNN and traditional classifier. The Hybrid-CS model fuses spatial and spectral information of the HSI in the form of multi-features combined with support vector machine learning. Experiments were performed using the proposed model on a tree species dataset of Tahe Forestry Bureau, and results were compared to those of the latest methods. Results indicate the superiority of our method. In particular, the Hybrid-CS model is more efficient than other deep learning network models and is more suited for tree species classification.
